# Research progress on immune aging and its mechanisms affecting geriatric diseases

**DOI:** 10.1002/agm2.12089

**Published:** 2019-12-08

**Authors:** Yanping Yu, Songbai Zheng

**Affiliations:** ^1^ Department of Geriatric Medicine Hua Dong Hospital Shanghai China

**Keywords:** immune aging, immune cells, immune organs, immunosenescence

## Abstract

*Immunosenescence*, also known as immune aging, refers to the degeneration, compensation, and reconstruction of the immune system with aging. Immune aging is an important factor in the increased susceptibility of the elderly to infectious diseases, malignant tumors, and a variety of chronic diseases and has long been a hotspot in geriatrics and immunology research. In this paper, the characteristics and progression of immune aging are briefly reviewed for clinicians' reference.

## 1 INTRODUCTION

The immune system consists of immune organs and tissues (bone marrow [BM], thymus, spleen, lymph nodes), immune cells (such as lymphocytes, monocytes/macrophages, dendritic cells, natural killer [NK] cells, and neutrophils) and immune molecules (such as immunoglobulins, complement, various membrane molecules, and cytokines). The BM and thymus are the central immune organs of the human body, being places where immune cells are present and where they differentiate, develop, and mature. Lymph nodes and the spleen are peripheral immune organs, being places where mature T and B cells settle and produce an immune response. Lymphocytes mainly mediate the adaptive immune response, while innate immune cells and molecules mainly mediate the innate immune response. Under physiological conditions, innate and adaptive immune responses are interdependent, and closely cooperate to complete a host's immune defense, immune surveillance, and immunologic homeostasis, resulting in immune protection of the body.

### 1.1 Bone marrow

BM is divided into red BM and yellow BM. Red BM makes red blood cells, white blood cells, and platelets. White blood cells can kill a variety of pathogens, including bacteria and viruses. Therefore, the BM is not only a hematopoietic organ, but is also an important immune organ that is the main site of a secondary immune response and antibody production. The amount of BM hematopoietic tissue decreases gradually with age and is replaced by adipose tissue.^1^

Hematopoietic stem cells (HSCs) are located in the specific microenvironment of the BM, where the potential for self‐renewal and differentiation is significantly influenced by senescence, as shown in human as well as animal studies.^1^ The regenerative activity and self‐renewal ability of HSCs in the elderly decrease and tend to differentiate along with the myeloid system.^2^ This eventually leads to a decrease of T and B lymphocyte production.^3^ Plasma cells in the BM have the ability to produce antibodies that are persistent and of high affinity, which is of vital importance in providing enduring immune protection.^4^ The percentage of plasma cells in the BM decreases with age, resulting in an impaired humoral immune response in the elderly. The total number of CD4+ and CD8+ T cells in the BM is not affected, but the size of CD4+ and CD8+ T cell subsets in the BM changes with age.^5^ Studies have shown that mesenchymal stromal cells in patients with multiple myeloma (B‐cell malignant tumors) exhibit aging characteristics: reduced phenotypic changes, differentiation and proliferation, and the production of higher levels of pro‐inflammatory cytokines. It is suggested that the BM microenvironment may be a key factor in the pathogenesis of this disease.^2^ In addition, an increase in the pro‐inflammatory factors tumor necrosis factor (TNF)‐α, interleukin (IL)‐6, and IL‐15 in the BM of the elderly can stimulate osteoclast bone resorption, which may be related to the susceptibility of the elderly to osteoporosis and osteoarthritis.^6^

### 1.2 Thymus

The thymus is the first organ that begins to age in the body. It weighs about 15‐20 g during the neonatal period, and then increases with age, reaching 30‐40 g at puberty.^7^ After this, age‐related atrophy occurs in the thymus, with its weight decreasing at a rate of 1%‐3% and finally dropping to 10‐15 g at 60 years of age.^8^ With aging, the tissue structure of the thymus also degenerates: in aged rats, the thymic cortex became thinner, the boundary between the skin and medulla was unclear, and the ratio of skin to medulla, number of cortical cells, and volume of cells decreased significantly. Cellular arrangement became sparse, the gap enlarged, the periphery of cells became blurred, several cells were apoptotic, and interstitial fat and fibrosis were obvious.^9^ Under an electron microscope, mitochondria in thymic cortical cells of aged rats swelled, the cristae decreased and became irregular or scarce, or dissolved into vacuoles, other organelles degenerated and decreased, and many crumpled processes were formed on the surface of thymic cortical cells. However, the mitochondria in the cytoplasm of epithelial reticular cells were swollen and denatured, the cristae partially disappeared, and tensin fibers and vacuoles were significantly increased.^9^ The degeneration of aging thymic cells can directly lead to a development disorder of T lymphocytes in the thymus and a decrease in the number of T lymphocytes exported to the periphery,^10^ which leads to the weakening of a T‐cell‐mediated immune effect. This is closely related to infection, autoimmune diseases, tumors, and other diseases.^11^ It has been shown that chronic inflammation is magnified during aging by the release of autoreactive T cells that infiltrate and produce inflammatory responses during thymic atrophy.^12^

### 1.3 Spleen

The spleen is the largest peripheral lymphoid organ in the human body; it consists of red and white pulp, and has hematopoietic and hemofiltration functions. It is also an important location for an immune response and immune effector molecules after lymphocyte migration and antigen stimulation. In aged rats, the white pulp in the spleen decreased gradually, the structure became disordered, the density of lymphocytes decreased, the boundary between red and white pulps became unclear,^13^ the number of macrophages increased, and the amount of hemosiderin increased.^14^ It is believed that the increase in macrophages of the spleen is due to an increase in aging and death components in the blood during aging; macrophages in the spleen are strongly phagocytic, leading to the removal of aging and dying autologous cells in the blood. As a result, the spleens of aged mice contain many macrophages.^15^ B cells in the marginal zone constantly collect complement conditioning antigens in the blood and deliver them to follicular dendritic cells (FDCs) in B lymphoid follicles that then migrate back to the marginal zone (MZ).^16^ Studies have shown that the localization and shuttling of B cells between MZ and B lymphoid follicles in the spleen of the elderly were significantly impaired, while B‐cell deficiency in the MZ was associated with an increased risk of pneumococcal infection and a weakened antibody response to microbial capsular polysaccharides. These results suggest that aging affects the ability of B cells in the MZ to obtain antigens and subsequently produce an effective TI‐antibody response.^17^ FDCs can promote a B‐cell‐mediated response by maintaining germinal centers and promoting the production of high‐affinity antibodies.^2^ The decrease in FDC density in the aging spleen leads to an impaired ability to capture and retain immune complexes, form germinal centers, and produce antibodies.^2^ With aging, these structural changes in the spleen affect the function of immune cells so as to eventually lead to degeneration in the efficiency of the immune response.

### 1.4 Lymph node

Lymph nodes are places where antigens are stimulated and immune responses are produced. The total number of lymph nodes in the human body ranges from 300 to 500, the total weight of which is about 100 g.^18^ The number of lymph nodes in the human body decreases with age; lymphoid tissue in the cortex and medulla of lymph nodes decreases, and fat deposits gradually become transparent.^19^ This transparency of lymph nodes impairs their ability to filter malignant cells or microorganisms. Consequently, this allows pathogens and malignant tumor cells an opportunity to spread. In a study of cervical lymph node metastasis in oral squamous cell carcinoma, it was found that the cervical lymph node metastasis rate was 33.3% in a younger age group (≤60 years) and 43.1% in an older age group (>60 years; *P* ≤ 0.013), suggesting cervical lymph node metastasis in oral cancer increases with age.^20^ Hyaline lymph nodes may also be one of the causes of increasing metastasis of malignant tumors.^19^ Studies have shown that the number and volume of germinal centers in lymph nodes decrease with age, resulting in a decrease in their reactivity.^21^ However, it has been suggested that the migration of B cells in aging lymph nodes, as well as the ability to obtain immune complexes or produce immunoglobulin do not appear to be impaired. The number of FDCs in lymph nodes of the elderly was significantly decreased^22^; the expression of the chemokine CXCL13 was also decreased, and the ability to retain immune complexes was significantly impaired. Such defects of FDCs are potential causes for the poor humoral immunity observed in the elderly.^22^ Subcapsular sinus macrophages collect pathogenic substances and antigens from lymph nodes and deliver them to B cells and FDCs, also within lymph nodes. Studies have shown an increase in the number of subcapsular sinus macrophages and other macrophages in the lymph nodes of the elderly. However, the uptake of immune complexes did not change with age.^22^

## 2 IMMUNE CELLS AND IMMUNE MOLECULES

### 2.1 T cells and related immune molecules

A significant characteristic of T cell proliferation and aging is a decrease in proliferative ability (Table 1), which is closely related to the expression of CD57 on the T cell surface,^23^ the shortening of telomere length, and a decrease in telomerase activity.^24^ CD57+ T cells cannot proliferate after antigen stimulation *in vitro* and are highly sensitive to activation‐induced apoptosis,^25^ compared with CD28+ CD57− T cells. CD57+ T cells produce more pro‐inflammatory cytokines and exert greater cytotoxicity.^26^ Cohen et al^27^ confirmed that the shorter the telomere of T cells, the higher the probability of infection, especially in CD8+ CD28− T cells; the correlation between telomere shortening and infection rate increased with age. The decrease in proliferation by aging T cells may lead to the delayed clearance of pathogens and prolonged duration of infection.

**Table 1:** Age‐related changes in the adaptive immune system

Cell type	Age‐related increase	Age‐related decrease
T cells	Memory T cells↑	Proliferation ability↓
	CD8+ CD28− T cells↑	Naive T cells↓
	Memory Treg cells↑	
B cells	Memory B cells↑	Naive B cells↓
	IL‐1α, IL‐1β, IL‐6 and TNF‐α↑	The mutation frequency
	Immunoglobulin in the circulation↑	Antibody diversity and affinity↓

Abbreviations: IL, interleukin; TNF, tumor necrosis factor; Treg cells, regulatory T cells.

One of the hallmarks of immune aging is a decrease in the proportion and number of peripheral initial T cells and an increase in the number of memory T cells (Table 1).^28^ This change has been identified as one of the main reasons for the increase in infection and cancer incidence in the elderly.^29^ Recent studies have shown that the accumulation of CD8+ memory T cells is mainly the result of persistent antigen stimulation caused by herpesvirus (especially cytomegalovirus) infection.^30^ Another sign of T cell senescence is the decreased expression of CD28 molecules. With aging, it was observed that the proportion of CD28− T cells increased gradually, especially in CD8+ T cell subsets (Table 1).^31^ CD8+ CD28− T cells show oligoclonal amplification.^32^ Although cloned cells may function and mediate protective immunity against virus reattack, their presence results in impaired T cell receptor diversity and a narrower antigen recognition spectrum, thus significantly reducing the response to new pathogens. CD4+ CD28− T cells can not only directly kill target cells by secreting perforin and granzyme, causing local inflammatory lesions, but also activate macrophages by secreting interferon (IFN)‐γ, promote the formation of foam cells, and lead to the formation and development of atherosclerotic plaques.^26^

Regulatory T cells (Tregs) are a subset of T cells that inhibit autoreactive effects. The anti‐inflammatory effects of cytokines released by Tregs (such as IL‐10 and transforming growth factor β) on other immune cells were inhibited or released in a cell‐to‐cell contact‐dependent manner.^33^ Van der Geest et al showed that memory Treg cells increased in CD4+ T cell subsets in the elderly (Table 1), and the number of memory Treg cells in circulation negatively correlated with a humoral immune response after vaccination in the elderly.^34^ It has also been suggested that an increase in Treg cells may be essential for the prevention of aging‐related autoimmune diseases, such as rheumatoid arthritis.^35^ Yang et al^36^ also found that the expression rate of Treg cells in the peripheral blood of patients with systemic lupus erythematosus (SLE) was significantly decreased, especially in an active stage. Patients with SLE are treated by inducing remission with glucocorticoid and/or cyclophosphamide. It was found that the ratio of Treg cells in the blood of such patients increased after treatment. In patients with SLE, the disease activity index decreased significantly, the proportion of Treg cells correlated well with the lupus activity index, and Tregs could effectively inhibit the occurrence of SLE nephropathy.^37^

### 2.2 B cells and related immune molecules

Min et al^38^ found that with physiological aging of the body, the number of lymphoid stem cells decreased while the ability to differentiate into progenitor B cells decreased significantly (Table 1), resulting in a decrease in the number of progenitor B cells with aging.^39^ The percentage and absolute number of B cells decreased, while the peripheral level remained unchanged.^40^ With an increase in age, the mutation frequency of B cells decreased (Table 1), which resulted in a decrease in proliferation and differentiation of peripheral B cells.^41^ The sensitivity of memory B cells to apoptosis also decreased in the elderly, leading to specific cloning and the amplification of certain B cells. Such expansion limits the diversity of the B cell bank and affects the efficacy of vaccination in older individuals.^42^ In addition, memory B cells can produce high levels of pro‐inflammatory cytokines, such as IL‐1α, IL‐1β, IL‐6, and TNF‐α (Table 1),^43^ suggesting that such cells may be involved in an increase in chronic inflammatory diseases in the elderly.

In elderly patients, although the number of B cells decreased, the level of immunoglobulins in the circulation increased (Table 1).^44^ Previous studies have shown that IgM and IgD levels negatively correlated with age, while IgG and IgA correlated in a positive manner, indicating a decrease in the B cell bank that can be used to respond to new antigen attacks in the elderly.^45^ Because IgM memory B cells are involved in the response to *Streptococcus pneumoniae* infection, a decrease in IgM may be the reason for an increased susceptibility to *S. pneumoniae* in the aged.^43^ In addition, many defects in the development of B cells occur in the elderly, which lead to a decrease in antibody diversity and affinity (Table 1).^23^ Data from a study of B cells obtained after influenza vaccination in the elderly (≥65 years) showed that their antibody response levels (hemagglutination inhibition test) decreased by 75% compared with young people, mainly reflected in high‐affinity IgG antibody.^46^

B cells can be sorted into B1 and B2 cells according to their origin. B2 cells produce single reactive antibodies against foreign antigens, while B1 cells produce antibodies with low affinity, such as IgM, with multiple reactivity that can form a variety of autoantibodies. B1 cells play an important role in autoimmune diseases mediated by antibodies (such as SLE, rheumatoid arthritis, and Graves' disease).^47^ The shift of B‐cell subsets to CD5+ B1 in aging individuals may be one of the reasons why the elderly are prone to autoimmune diseases.^41^

### 2.3 Macrophages and related immune molecules

Macrophages are derived from monocytes to tissue differentiation and maturation. Being strongly phagocytic, and having the ability to process and present antigens, macrophages are an important part of the body's immune system. The sensitivity of macrophages to IFN‐γ was shown to be significantly weakened in aged animals. Stimulated by a saturating IFN‐γ level, the expression of major histocompatibility II molecules on the surface of macrophages from aged mice was only half of that of young mice.^48^ In addition, the level of prostaglandin (PGE) 2 secreted by activated macrophages in the elderly was found to be significantly higher than that in young people (Table 2). PGE2 inhibited the expression of major histocompatibility II and the production of IL‐12 on the cell surface, resulting in a decrease in antigen presentation by macrophages with age.^49^ A high level of PGE2 also inhibited the expression of HLA‐DR (MHC class II molecule) on the surface of tumor cells, which is conducive to tumor escape from immune surveillance, thus allowing tumor growth and metastasis.^50^ Other aging characteristics of macrophages include decreased toll‐like receptor (TLR) expression,^51^ decreased superoxide anion production,^49^ and impaired phagocytosis and chemotaxis (Table 2).^52^ The interaction between TLR and pathogens stimulates the secretion of broad‐spectrum antimicrobial peptides to destroy pathogens and trigger inflammatory reactions. Studies have shown that in the context of human aging, TLR function is weakened. The TLR signaling pathway becomes dysfunctional, showing abnormal and persistent activation, which may lead to an increase in the incidence and death from infectious diseases in elderly patients.^53^ With an increase in age, the expression and function of TLR became impaired, and the production of the macrophage pro‐inflammatory cytokines, TNF‐α, IL‐6, and IL‐1β, decreased (Table 2).^54^ The damage from delayed‐type hypersensitivity in the elderly is related to a decrease in TNF‐α produced by skin macrophages.^54^ In addition, chemotaxis and phagocytic activity by macrophages from aged mice were decreased, and the number of macrophages infiltrating wounds was also decreased, which led to the delayed removal of debris from the injured site and hindered the wound‐healing process.^55^

**Table 2:** Age‐related changes in the innate immune system

Cell type	Age‐related increase	Age‐related decrease
Macrophages	PGE2 production	Chemotaxis
		Phagocytosis
		TLR expression and function
		Cytokine production
Neutrophils	No change in the number of cells	Chemotaxis
		Phagocytosis
		Superoxide production
DC		IFN‐I/III production
		Antigen presentation
		Chemotaxis and endocytosis
NK cells	Total number of cells	Proliferative response to IL‐2
		Migration ability

Abbreviations: DC, dendritic cells; IFN, interferon; IL, interleukin; NK, natural killer; PGE2, prostaglandin 2; TLR, toll‐like receptor.

### 2.4 Neutrophils and related immune molecules

Neutrophils constitute the main immune defense against rapidly dividing bacteria, yeast, and fungi in infections. These cells reduce infections by phagocytosis, the production of reactive oxygen species (ROS) and nitrogen substances, and the release of proteolytic enzymes and antibacterial peptides in cytoplasmic granules.^56^ The number of neutrophils remained unchanged with age (Table 2), but the function of neutrophils decreased due to abnormal signal transduction pathways,^49^ including chemotaxis, phagocytosis,^44^ production of ROS (Table 2), intracellular killing, and degranulation.^57^ Reduced chemotaxis by neutrophils in the elderly means that these cells take longer to reach the site of an infection compared to those in younger patients, thereby increasing the risk of infection for such patients. In addition to chemotactic defects, neutrophils from elderly hosts also show impaired pathogen clearance. *S. pneumoniae* is an important pathogen associated with high morbidity and mortality in the elderly. It can cause a variety of infections, from mild upper respiratory tract infections to serious life‐threatening diseases, such as pneumonia, bacteremia, and meningitis.^58^ In the elderly, the functional activity of anti‐*S. pneumoniae* antibody and the ability of neutrophils to phagocytose and regulate *S. pneumoniae* were impaired; the production of ROS was also reduced, which led to a decrease in the phagocytosis and killing of *S. pneumoniae*. At the same time, the migration of neutrophils to the lungs was decreased in older mice, which increased the risk of pulmonary infection and recurrence.^59^ A neutrophil extracellular bactericidal network (neutrophil extracellular traps [NETs]) is a way to capture pathogens. NETs can promote the killing of pathogens by increasing the contact between pathogens and antimicrobial proteins, thereby preventing bacteria from invading the bloodstream.^60^ Compared with young mice, the number of NETs in a skin infection site containing *Staphylococcus aureus* in old mice was lower, which partly promoted the spread of *S. aureus* into the bloodstream and distal organs.^61^

### 2.5 Dendritic cells and related immune molecules

Dendritic cells are a type of antigen‐presenting cell that have attracted much attention in recent years. It is the strongest antigen‐presenting cell in the immune system and initiates a T‐cell‐mediated immune response. However, the number of dendritic cells in the peripheral blood, skin, and thymus of the elderly was found to be reduced.^46^ Plasmacytoid dendritic cells (pDCs) can produce IFN‐I/III, which is particularly important for host defense against pathogens, especially viral invasion. However, the number of pDCs and their ability to produce IFN‐I and IFN‐III in the circulation of the elderly were significantly reduced (Table 2)^62^; the ability to present antigens to CD4+ T cells and CD8+ T cells was also decreased (Table 2).^63^ These age‐related changes in pDCs may lead to an impaired immune defense against viral infection in the elderly. Myeloid dendritic cells (mDCs) show the basic functions of phagocytosis, chemotaxis (Table 2), and the ability to produce IL‐12; however, these are impaired in the elderly. The ability of mDCs to initially activate CD4+ T cells by presenting antigens is also reduced,^63^ which may lead to a low response to vaccines and increased susceptibility to infection in the elderly. In addition, basic levels of IL‐6 and TNF‐α in mDCs, and basic levels of IFN‐γ and TNF‐α in pDCs were increased in the elderly,^44^ while chronic low‐grade inflammation (slight increase of inflammatory factors, such as IL‐6 and TNF‐α), atherosclerotic cardiovascular and cerebrovascular diseases, Parkinson's disease, Alzheimer's disease, and other neurodegenerative diseases are closely related to the occurrence of malignant tumors.^64^

### 2.6 NK cells and related immune molecules

Rapid lytic activity or the direct production of cytokines, such as IFN‐γ and TNF‐α, of NK cells play an important role in the host defense against invasive pathology.^65^ The absolute number of NK cells in the elderly increased (Table 2), specifically, CD56^bright^ NK cell subsets decreased and mature CD56^dim^ NK cell subsets increased.^24^ Although the number of NK cells increased in the elderly, the cytotoxicity of NK cells decreased in terms of the level of cytokines and chemokines produced by a single cell.^66^ Studies have shown that high NK cytotoxicity is associated with longevity and health, while low NK cytotoxicity is associated with infection, increased morbidity and mortality from atherosclerosis, and poor responsiveness to influenza vaccines.^59^ In addition, the reduced toxicity of NK cells can also increase the risk of cancer. An 11‐year prospective study of 3500 middle‐aged and elderly people showed an increased incidence of cancer in patients with low initial NK cytotoxicity.^67^ It has been observed that the level of IFN‐γ produced by NK cells in the elderly and stimulated by IL‐2 or IL‐12 decreased (Table 2),^44^ and migration of NK cells decreased with age (Table 2).^5^ After a virus attacks the body, NK cells collect in a number of draining lymph nodes. This age‐related decrease in NK cells leads to an increase in susceptibility to a virus.^68^

The mechanism of immune aging is extremely complex and closely related to inflammatory aging. It has become a significant but difficult area of geriatric research in recent years. From the existing research data, immune aging is very prominent, and plays an important role in the occurrence and development of infectious diseases, malignant tumors, and a variety of diseases in the elderly. This article has focused on the characteristics of aging and its relationship with the clinic. It has attempted to provide reference information for clinicians, especially geriatricians, in order to understand the clinical significance of immune aging. It is expected that more stable and reliable indicators may be used in the assessment of the immune status and in immunotherapy for elderly patients. However, the existing literature indicates that more animal experimental studies in comparison with clinical studies have been performed in immune aging. There is still a long way to go to achieve the above goals, with more clinically oriented, in‐depth research needed.

## CONFLICTS OF INTEREST

There are no conflicts of interest to be reported by the authors of this study.

## AUTHOR CONTRIBUTIONS

Songbai Zheng is responsible for proposing research propositions and revised final versions. Yanping Yu is responsible for collecting, organizing, and analyzing data and writing the manuscript.
